# Nomograms Predict Overall Survival and Cancer-Speciﬁc Survival in Patients with Fibrosarcoma: A SEER-Based Study

**DOI:** 10.1155/2020/8284931

**Published:** 2020-09-26

**Authors:** Guang-Heng Xiang, Juan-Juan Zhu, Chen-Rong Ke, Yi-Min Weng, Ming-Qiao Fang, Si-Pin Zhu, Yu-An Li, Jian Xiao, Lei Xu

**Affiliations:** ^1^Department of Orthopaedic, The Second Affiliated Hospital and Yuying Children's Hospital of Wenzhou Medical University, Wenzhou, Zhejiang 325035, China; ^2^School of Pharmaceutical Sciences, Wenzhou Medical University, Wenzhou, Zhejiang 325035, China; ^3^Department of Geriatrics and Neurology, The Second Affiliated Hospital and Yuying Children's Hospital of Wenzhou Medical University, Wenzhou, Zhejiang 325035, China; ^4^Department of Pediatric Surgery, The Second Affiliated Hospital and Yuying Children's Hospital of Wenzhou Medical University, Wenzhou, Zhejiang 325035, China

## Abstract

**Purpose:**

Due to the rarity, it is difficult to predict the survival of patients with fibrosarcoma. This study aimed to apply a nomogram to predict survival outcomes in patients with fibrosarcoma.

**Methods:**

A total of 2235 patients with diagnoses of fibrosarcoma were registered in the Surveillance, Epidemiology, and End Results database, of whom 663 patients were eventually enrolled. Univariate and multivariate Cox analyses were used to identify independent prognostic factors. Nomograms were constructed to predict 3-year and 5‐year overall survival and cancer‐specific survival of patients with fibrosarcoma.

**Results:**

In univariate and multivariate analyses of OS, age, sex, race, tumor stage, pathologic grade, use of surgery, and tumor size were identiﬁed as independent prognostic factors. Age, sex, tumor stage, pathologic grade, use of surgery, and tumor size were significantly associated with CSS. These characteristics were further included to establish the nomogram for predicting 3-year and 5-year OS and CSS. For the internal validation of the nomogram predictions of OS and CSS, the *C*-indices were 0.784 and 0.801.

**Conclusion:**

We developed the nomograms that estimated 3-year and 5-year OS and CSS. These nomograms not only have good discrimination performance and calibration but also provide patients with better clinical benefits.

## 1. Introduction

Fibrosarcoma (FS) refers to a specific entity, which is by definition a diagnosis of exclusion, on the basis of the WHO classification of tumors of soft tissue and bone [1]. Fibrosarcoma belongs to the sarcoma cancer group and is a rare and highly malignant tumor of mesenchymal origin. Fibrosarcoma can occur in any anatomical location. It does not directly produce bone or cartilage but forms a primary or secondary bone tumor [2]. Central fibrosarcoma is caused by fibrous tissue in the medullary canal, but periosteal fibrosarcoma results from periosteal connective tissue [3]. Fibrosarcoma includes infant (congenital) fibrosarcoma and adult fibrosarcoma, and adult fibrosarcoma is defined as “malignant neoplasm composed of fibroblasts with variable collagen production and, in classical cases, a “herringbone” architecture” [1]. The prognosis of adult fibrosarcoma is much worse than infant fibroids. In the past period, the incidence of FS has dropped dramatically, and recent data indicate that it accounts for only 3.6% of sarcomas [4]. Surgery is the standard treatment of patients with localized FS. In addition, adjuvant chemotherapy, radiotherapy, and/or hyperthermia can be discussed in some cases [5].

As with other sarcomas, the overall prognosis of fibrosarcoma depends on tumor size, tumor nature, tumor grade, and the presence or absence of distant metastases [1, 6–9]. The prognostic factors associated with survival are numerous and complex, and it is meaningful to establish a model to accurately predict the survival outcome of FS. Prognostic nomograms have proven to be an effective method for accurately predicting survival outcomes in cancer patients such as lung cancer, rectal cancer, and gastric cancer patients [10–12]. However, to the best of our knowledge, there is no report on the use of nomograms to predict FS survival outcomes. Therefore, the goal of our study is to identify independent factors that influence the overall survival (OS) and cancer-specific survival (CSS) of patients with FS and then establish a nomogram to accurately predict the incidence of OS and CSS at 3-year and 5-year.

## 2. Materials and Methods

### 2.1. Patients Source and Selection

The Surveillance, Epidemiology, and End Results (SEER) registry program provides information on cancer statistics since 1973. This database covering approximately 28% of the population in 18 different regions of the United States is supported by the Surveillance Research Program in National Cancer Institute Division of Cancer Control and Population Sciences. The SEER∗Stat software Version 8.3.6 (https://seer.cancer.gov/seerstat/, NCI, Bethesda, USA) provides a convenient, intuitive mechanism for the analysis of SEER and other cancer-related databases.

The inclusion criteria for this study were as follows: (a) patients were diagnosed as 8811/3, 8812/3, 8813/3, and 8814/3 in ICD-O-3 (International Classification of Disease for Oncology, 3rd Edition); (b) histological examination confirmed as FS; (c) completed the follow-up period; and (d) complete survival and death date. Exclusion criteria in this study were as follows: (a) only autopsy or death certificate; (b) the data of tumor size, pathologic grade, or stage missing; and (c) the data of surgery treatment missing.

### 2.2. Patients Variables

The information about clinicopathological features, including age at diagnosis, year of diagnosis, race, sex, histology, pathologic grade, disease stage, tumor size, use of surgery, marital status, vital status, and months of survival, were recorded in our study. The race was divided into white, black, and others. Marital status was categorized as married and unmarried. The pathological grade was divided into four categories according to the “ICD-O-3 grade”: grades I, II, III, and IV. According to the American Joint Committee on Cancer (AJCC) staging system, the FS tumor stage was divided into local, regional, and distant (Supplementary Figure S1).

### 2.3. Statistical Analysis

**Figure 1:**
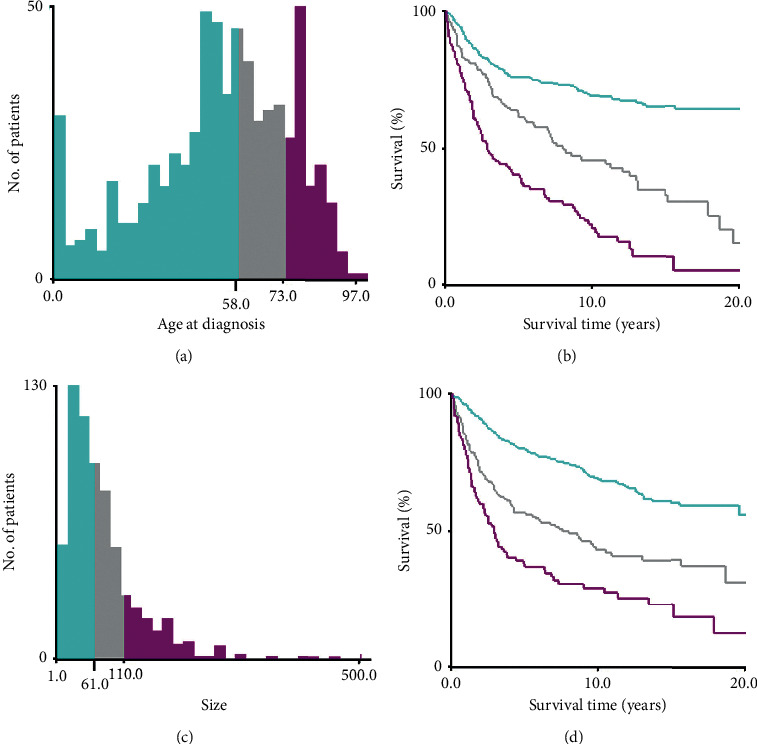
Identiﬁcation of optimal cuto values of age of diagnosis and tumor size via *X*-tile analysis. Optimal cuto values of age were identiﬁed as 58 and 73 years based on overall survival (a). Optimal cuto values of tumor size were identiﬁed as 61 mm and 110 mm based on overall survival (c). Histogram and Kaplan–Meier analysis were developed based on these cuto values (b) and (d).

SPSS 23.0 (IBM Corp.) was used to assess the patient variables collected from the SEER databases. The two main endpoints of this study were overall survival and cancer-specific survival. Overall survival was defined as the period from diagnosis to death from any disease cause. Cancer-specific survival was defined as the period from diagnosis to death from FS. The cuto value of age at diagnosis and tumor size was calculated by *X*-tile software (Yale University, New Haven, Connecticut, USA), according to OS (Figure 1). We constructed the cumulative survival curves using the Kaplan–Meier method and compared the variables by a log-rank test. Cox proportional hazard regression analyses were used to determine independent prognostic factors, and the results are presented as hazard ratios and corresponding 95% confidence intervals (95% CIs). According to the results obtained by multivariate Cox proportional hazards regression analyses, nomograms for 3-year and 5-year OS and 3-year and 5-year CSS were constructed by applying the rms package in *R* software, version 3.6.1 (http://www.r-project.org/). Concordance index (*C*-index) was used to evaluate the reliability of nomogram [13]. The *C*-index ranged from 0.5 (a poor model) to 1.0 (a perfect model), and it more than 0.7 represented a good predictive performance [14]. The calibration curve was used to compare the conformity between the predicted and actual survival. A two-tailed *P* < 0.05 was considered statistically significant.

## 3. Results

A total of 2235 patients diagnoses as FS were enrolled in the SEER database from 1975 to 2016, among them 663 patients eventually participated in our study on the basis of the above inclusion and exclusion criteria. The clinical basic characteristics of patients are shown in Table 1. Among these 663 cases, 331 patients were female (49.9%) and 332 patients were male (50.1%). The average age of the whole population was 52.65 years. The majority (78.7%) of tumors was found in white patients. With regard to the pathologic grades, grade II was the most common (*n* = 222 (33.5%)), followed by grade I (*n* = 156 (23.5%)), grade IV (*n* = 153 (23.1%)), and grade III (*n* = 132 (19.9%)). About the tumor size of FS, 53.4% of patients had a tumor size less than 61 mm, 27.7% had a tumor between 61 mm and 110 mm, and 18.9% had a tumor greater than 110 mm. Regarding tumor staging, 62.3% of patients had localized disease, 28.4% patients had regional disease, and the remaining 9.3% patients had distant disease. For treatment, 93.5% underwent surgery.

**Figure 2:**
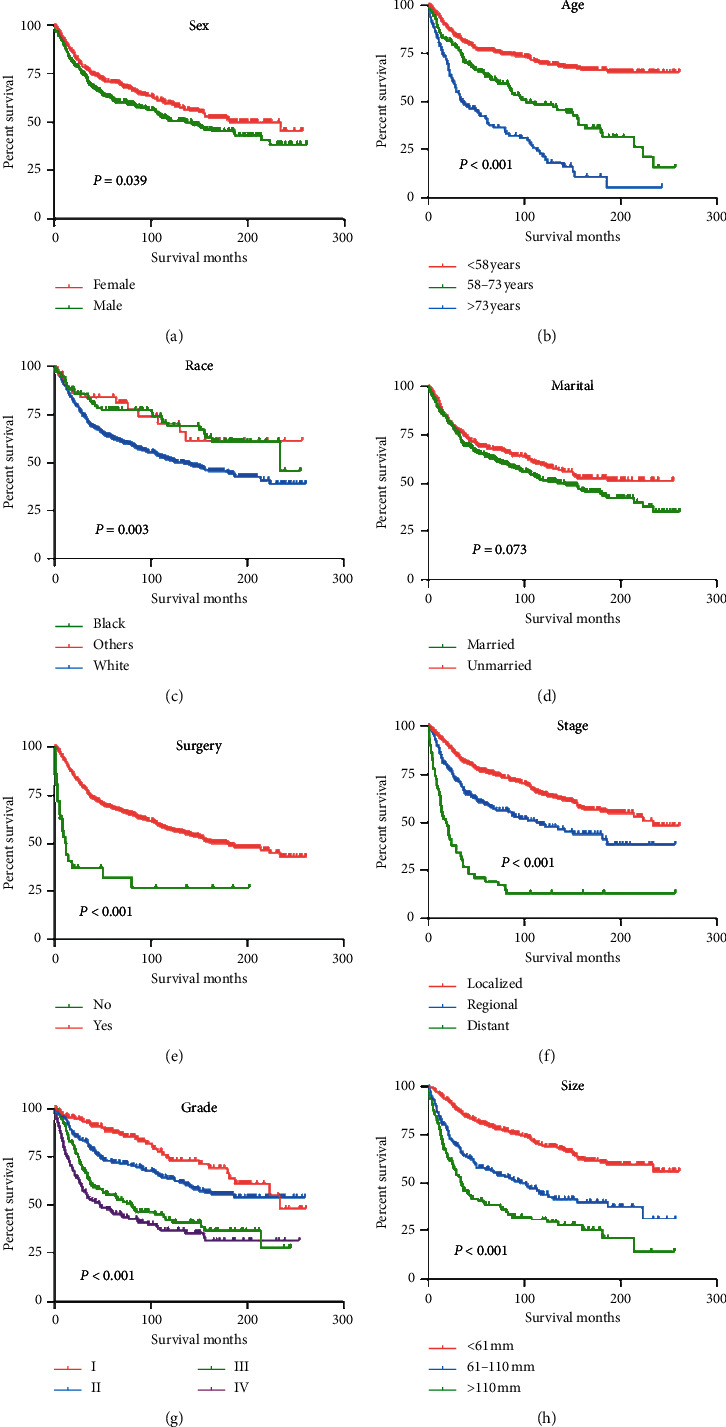
Kaplan–Meier curves of variables were performed for OS in patients with fibrosarcoma. (a) Sex, (b) age, (c) race, (d) marital, (e) use of surgery, (f) tumor stage, (g) tumor grade, and (h) tumor size.

As shown in Table 2, univariate and multivariate analyses of variables were performed for OS in patients with fibrosarcoma. There were seven variables involving patient age (*P* < 0.001), sex (*P*=0.039), race (*P*=0.003), tumor stage (*P* < 0.001), pathologic grade (*P* < 0.001), use of surgery (*P* < 0.001), and tumor size (*P* < 0.001) that were related to OS, and the marital had no significant difference (Figure 2). Further multivariate analyses of important factors identified by univariate analyses were performed, and the results showed that patient age, sex, surgery, tumor stage, pathologic grade, and tumor size were the independent risk factors.

**Figure 3:**
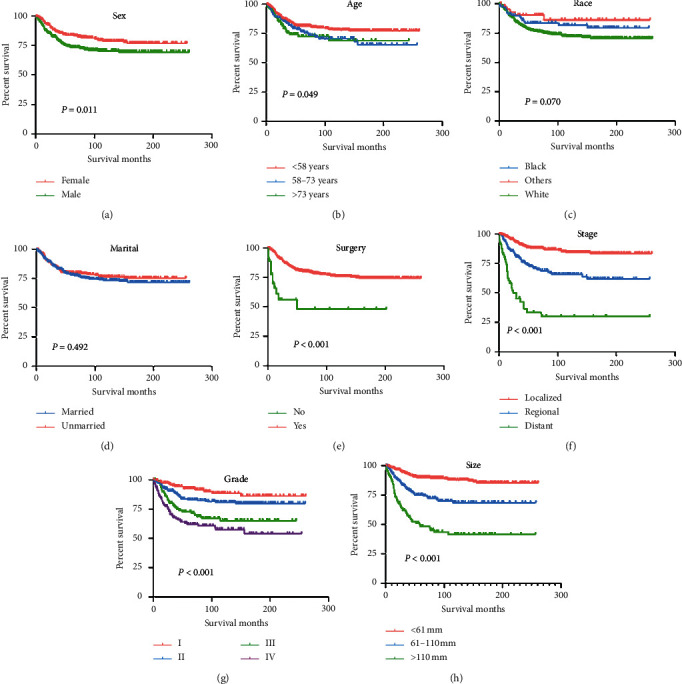
Kaplan–Meier curves of variables were performed for CSS in patients with fibrosarcoma. (a) Sex, (b) age, (c) race, (d) marital, (e) use of surgery, (f) tumor stage, (g) tumor grade, and (h) tumor size.

Univariate and multivariate analyses of variables associated with CSS of fibrosarcoma are shown in Table 3. The results of the analyses showed that patient age (*P*=0.049), sex (*P*=0.011), tumor stage (*P* < 0.001), pathologic grade (*P* < 0.001), use of surgery (*P* < 0.001), and tumor size (*P* < 0.001) were significantly associated with CSS, while race (*P*=0.070) and marital (*P*=0.492) were not significantly correlated with CSS (Figure 3). For the multivariate analyses results, sex, surgery, tumor stage, pathologic grade, and tumor size were determined as independent prognostic factors for CSS.

**Figure 4:**
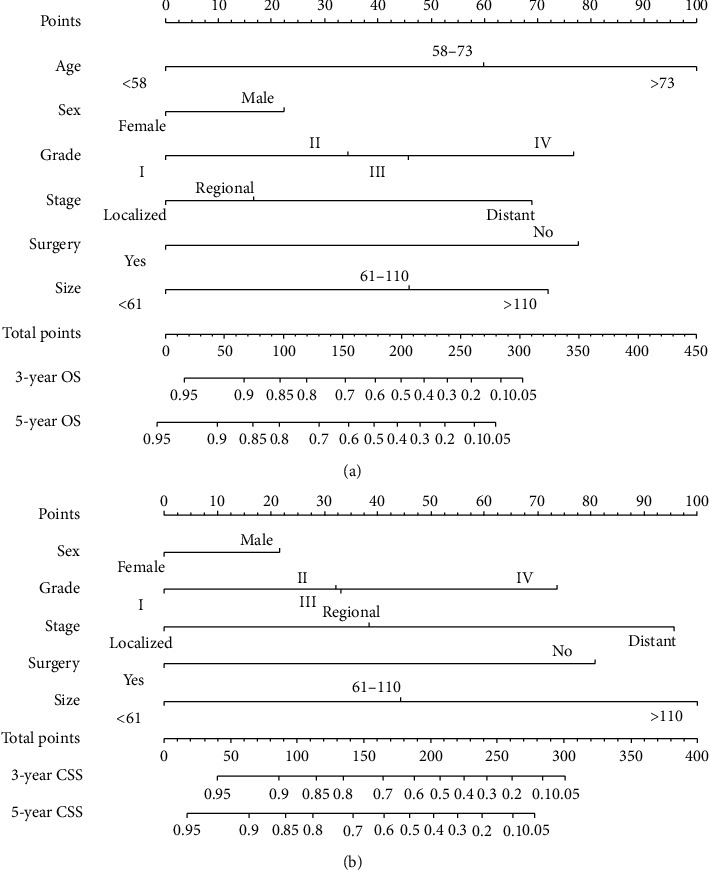
Nomogram to predict overall survival (OS) and cancer-specific survival (CSS) in patients with fibrosarcoma. (a) Predicting 3-year and 5-year OS rates. (b) Predicting 3-year and 5-year CSS rates.

**Figure 5:**
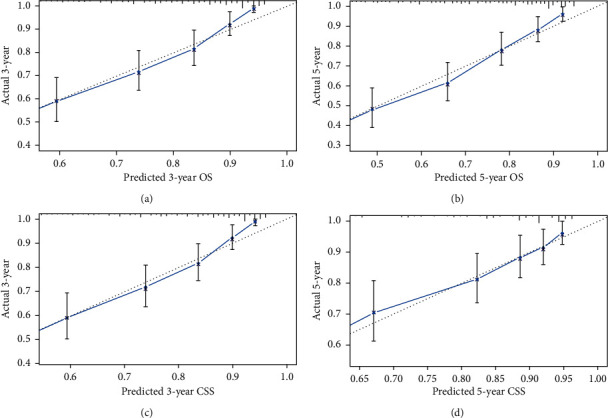
Calibration curves of the nomogram predicting overall survival (OS) and cancer-specific survival (CSS) in patients with fibrosarcoma. (a) 3-year OS rate, (b) 5-year OS rate, (c) 3-year CSS rate, and (d) 5-year CSS rate.

Independent risk factors determined by multivariate analyses were used to construct the prognostic nomograms for predicting 3-year and 5-year overall survival and cancer-specific survival of patients with fibrosarcoma (Figure 4). The result of OS prediction by nomogram showed that age was the main factor affecting prognosis, followed by surgery, pathological grade, tumor size, tumor stage, and gender, whereas as for CSS, the result showed that tumor size was the most critical factor affecting prognosis, followed by tumor stage, surgery, pathologic grade, and sex. For the internal validation of the nomograms of OS and CSS, the *C*-indices were 0.784 and 0.801, separately. The calibration plots showed the excellent consistency between the nomogram prediction and the actual survival (Figure 5).

## 4. Discussion

Multiple prognostic factors can affect the survival outcome of cancer patients, while a single prognostic factor cannot fully predict the individual survival. In the light of the scarcity and differences in fibrosarcoma cases, assessing clinical prognosis outcomes can be very challenging. Relying on the traditional AJCC staging system as before has not been sufficient to accurately guide treatment and assess the prognosis of cancer. Nomogram is a graphical illustration of a statistical model for calculating the cumulative effect of several variables and can be used to predict individual survival outcomes. Nomograms have been established for a variety of cancers and have shown more accurate in predicting prognosis than traditional tools [14–19]. As far as we know, the present study is the first article to develop and validate the prognostic nomograms for both OS and CSS in patients with FS. We developed comprehensive nomograms for 3-year and 5-year overall survival and cancer-specific survival on the basis of 663 cases extracted from the SEER database.

Our results showed that the following independent prognostic factors could influence the survival of patients with FS: age, sex, surgery, tumor stage, pathologic grade, and tumor size. The result of OS prediction by nomogram showed that age was the main factor affecting prognosis. It was widely believed that age was related to the survival outcome of various cancers [20–23]. The correlation between age and OS might be partly due to our use of all-cause mortality rather than cancer-specific survival. In other words, older patients usually had chronic diseases or postoperative complications that made them more likely to die.

Although it appears that a larger tumor predicts a poor prognosis, it is necessary to conduct further studies to examine this. In previous studies, the effect of tumor size on survival was inconsistent. Most of these studies believed that larger tumors size was harmful to patient survival [24–27]. In contrast, other studies supported that tumor size had no influence on the survival [28–30]. Regarding our article, the result indicated that tumor size was the most important factor affecting CSS. One possible explanation for these findings was that tumor size during diagnosis was related to the treatment used, which might affect survival.

Multivariate COX regression analysis showed that sex, surgery, tumor stage, and pathologic grade were also independent prognostic indicators for FS patients. Gender was also an important variable related to the prognosis of patients with cancer. In our article, the survival rates of male FS patients were worse than that of female patients. Our study also showed that surgical treatment was related to a better prognosis. Tumor stage was also an independent prognostic factor. The presence of distant stage resulted with a lower survival rate than localized or regional stage. This trend further demonstrated the importance of improving early diagnosis. In addition, pathologic grade could reflect the biological behavior of malignant tumors, which are associated with the occurrence of distant metastasis, and worsened the prognosis of survival outcome.

This study was based on data extracted from the SEER database, which had a large sample size and sufficient cancer data. However, our research had some limitations. As a retrospective study, these findings may be needed to be further validated by randomized controlled trials and prospective studies. Some clinical pathological parameters, such as comorbidities, vascular infiltration, surgical margin status, chemotherapy, or other treatment, were not available in the SEER database, so we did not include these factors in the nomogram [31–34]. Finally, the *C*-index is a good nomogram verification tool, but it is more reliable if you use other independent large-scale datasets for external verification.

## 5. Conclusions

Patient age, sex, use of surgery, tumor stage, pathologic grade, and tumor size were determined as independent prognostic factors of patients with fibrosarcomas. We developed the nomograms that estimated 3-year and 5-year overall survival and cancer-specific survival based on 663 cases extracted from the SEER database. These nomograms not only had good discrimination performance and calibration but also provided patients with better clinical benefits.

## Figures and Tables

**Table 1:** Patient cohort characteristics.

Variables	Value, *n* (%)
Age, years	
<58	370 (55.8%)
58–73	158 (23.8%)
>73	135 (20.4%)

Sex	
Male	332 (50.1%)
Female	331 (49.9%)

Race	
White	522 (78.7%)
Black	92 (13.9%)
Others	49 (7.4%)

Surgery	
Yes	620 (93.5%)
No	43 (6.5%)

Marital status	
Married	345 (52.0%)
Unmarried	318 (48.0%)

Stage	
Localized	413 (62.3%)
Regional	188 (28.4%)
Distant	62 (9.3%)

Grade	
I	156 (23.5%)
II	222 (33.5%)
III	132 (19.9%)
IV	153 (23.1%)

Tumor size (mm)	
<61	354 (53.4%)
61–110	184 (27.7%)
>110	125 (18.9%)

**Table 2:** Univariate and multivariate analyses of variables associated with overall survival.

Variables	Univariate analysis	Multivariate analysis	
	*P* value	HR (95% CI)	*P* value
Age, years	<0.001		
<58		Reference	
58–73		2.200(1.622–2.986)	<0.001
>73		3.902 (2.911–5.230)	<0.001

Sex	0.039		
Male		Reference	
Female		0.756 (0.593–0.963)	0.024

Race	0.003		
White		Reference	
Black		0.814 (0.547–1.211)	0.310
Others		0.636 (0.361–1.121)	0.118

Surgery	<0.001		
Yes		Reference	
No		3.073 (1.993–4.738)	<0.001

Marital status	0.0726		
Married		Reference	
Unmarried		0.836 (0.651–1.075)	0.163

Stage	<0.001		
Localized		Reference	
Regional		1.244 (0.947–1.635)	0.117
Distant		2.660 (1.859–3.807)	<0.001

Grade	<0.001		
I		Reference	
II		1.655 (1.116–2.455)	0.012
III		1.907 (1.261–2.885)	0.002
IV		3.000 (2.023–4.450)	<0.001

Tumor size (mm)	<0.001		
<61		Reference	
61–110		1.931 (1.446–2.578)	<0.001
>110		2.784 (2.057–3.766)	<0.001

**Table 3:** Univariate and multivariate analyses of variables associated with cancer-specific death.

Variables	Univariate analysis	Multivariate analysis	
	*P* value	HR (95% CI)	*P* value
Age, years	0.049		
<58		Reference	
58–73		1.353(0.905–2.022)	0.140
>73		1.382 (0.892–2.139)	0.147

Sex	0.011		
Male		Reference	
Female		0.697 (0.494–0.984)	0.040

Race	0.070		
White		Reference	
Black		0.704 (0.411–1.206)	0.201
Others		0.466 (0.189–1.149)	0.097

Surgery	<0.001		
Yes		Reference	
No		3.470 (1.994–6.039)	<0.001

Marital status	0.492	
Married				
Unmarried				

Stage	<0.001		
Localized		Reference	
Regional		1.788 (1.206–2.650)	0.004
Distant		4.269 (2.656–6.860)	<0.001

Grade	<0.001		
I		Reference	
II		1.753 (0.956–3.215)	0.070
III		1.716 (0.921–3.198)	0.089
IV		3.321 (1.847–5.972)	<0.001

Tumor size (mm)	<0.001		
<61		Reference	
61–110		2.039 (1.301–3.195)	0.002
>110		4.579 (2.980–7.035)	<0.001

## Data Availability

Publicly available datasets were analyzed in this study. The data used to support this study are available at https://seer.cancer.gov/.

## Supplementary Materials

Supplementary Materials The study flow diagram of the selection process.
