# Supplementary figures and images for: Nomograms Predict Overall Survival and Cancer-Speciﬁc Survival in Patients with Fibrosarcoma: A SEER-Based Study
Source: J Oncol. 2020 Sep 26;2020:8284931. doi: 10.1155/2020/8284931 (PMC7533781; doi:10.1155/2020/8284931)

**Figure legends**

**SUPPLEMENTARY FIGURE** The study flow diagram of the selection process.


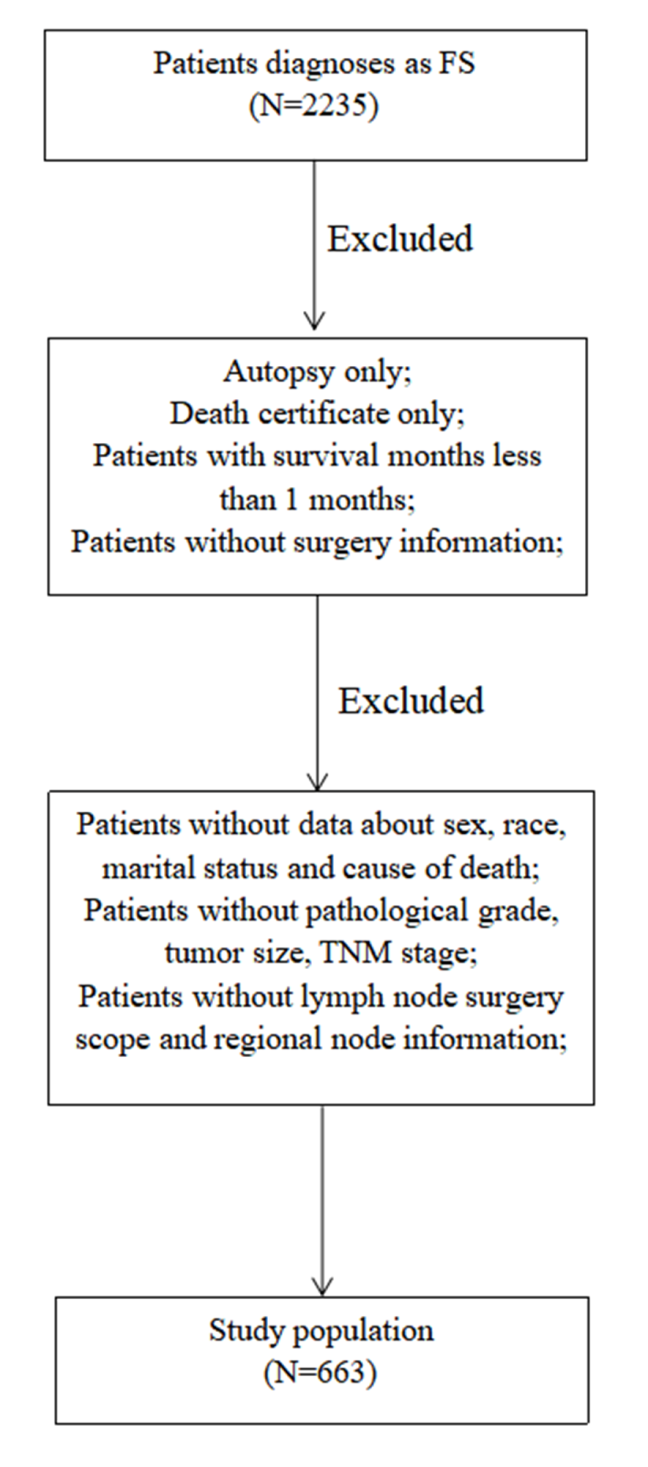

Supplement: Supplementary Materials — The study flow diagram of the selection process. [file 8284931.f1.docx]
